# Deep learning model for deep fake face recognition and detection

**DOI:** 10.7717/peerj-cs.881

**Published:** 2022-02-22

**Authors:** Suganthi ST, Mohamed Uvaze Ahamed Ayoobkhan, Krishna Kumar V, Nebojsa Bacanin, Venkatachalam K, Hubálovský Štěpán, Trojovský Pavel

**Affiliations:** 1 Department of Computer Engineering, Lebanese French University,Iraq., Erbil, Iraq; 2 Computing Department, Westminster International University in Tashkent, Tashkent, Uzbekistan; 3 Department of Computer Science Engineering, Sri Ramakrishna Engineering College, Coimbatore, India; 4 Department of Computing, Singidunum University, Belgrade, Serbia; 5 Department of Applied Cybernetics, Faculty of Science, University of Hradec Kralove, Hradec Kralove, Czech Republic; 6 Department of Mathematics, Faculty of Science, University of Hradec Kralove, Hradec Kralove, Czech Republic

**Keywords:** Deep fake, Fisherface, LBPH, DBN, RBM, Deep learning

## Abstract

Deep Learning is an effective technique and used in various fields of natural language processing, computer vision, image processing and machine vision. Deep fakes uses deep learning technique to synthesis and manipulate image of a person in which human beings cannot distinguish the fake one. By using generative adversarial neural networks (GAN) deep fakes are generated which may threaten the public. Detecting deep fake image content plays a vital role. Many research works have been done in detection of deep fakes in image manipulation. The main issues in the existing techniques are inaccurate, consumption time is high. In this work we implement detecting of deep fake face image analysis using deep learning technique of fisherface using Local Binary Pattern Histogram (FF-LBPH). Fisherface algorithm is used to recognize the face by reduction of the dimension in the face space using LBPH. Then apply DBN with RBM for deep fake detection classifier. The public data sets used in this work are FFHQ, 100K-Faces DFFD, CASIA-WebFace.

## Introduction

Digital manipulation of the face images include facial information of fake images using deep fake approaches (Korshunov & Marcel, 2018). In recent years, deepfake approach has become a popular technique in detecting fake images recently (Citron, 2019; Cellan-Jones, 2019). It is implemented by using deep learning technique in order to create fake images by swapping the face of one person by the face of another. In the year 2017, it was termed as Reddit user and it is commonly known as “deep fakes” using deep adversarial models (*i.e.*) Generative Adversarial Networks (GAN) and similarly, it transform the celebrity faces into porn videos (Maras & Alexandrou, 2019). The main issues in the fake pornography are including fake news in the content, financial fraud and hoaxes. At the same time, the advantages of deep fake is in the fields such as virtual reality, editing film and production. The chief working concepts of deep fakes are merging, replacing, combining and superimposing images using deep learning and machine learning techniques so as to create fake digital images or videos (Maras & Alexandrou, 2019).

Many software apps/tools are available through which deep fake images are created without a programming knowledge and technical side background information. Usually the profile pictures from the social media are taken and fake images or videos are developed with a help of the expert. Security enhancement in the detection of face swap and the accuracy are very low. To overcome these issues, this paper proposes a new strategy for detecting the deep fake facial images using the fisher face with an LBPH approach. In the digital image manipulation, techniques are applied in many fields which give more misinformation in the society. The scenarios such as creating fake news, providing false information in the political elections, create security threats (Allcott & Gentzkow, 2017; Lazer et al., 2018).

CNN based methods like XceptionNet, Meso Inception-Net, ResNet are used in the field of detecting deep fakes which include detection of visual artifacts, inconsistency in color during the time of performing blend operations in the image analysis. The process include identifying the forgery of face X-ray by blending the boundary forged image in the CNN model and classifying the loss in the detection of face X-ray (Li et al., 2020; Li & Lyu, 2018; Afchar et al., 2018; Dang et al., 2020). In the media, articles use the biometric technology for the detection of deep fakes. In order to detect the difference between real and fake images in the field of ocular biometric, CNN methods such as Squeeze Net, DenseNet, ResNet and light CNN are used  (Nguyen & Derakhshani, 2020). The main contributions of this research work are the following:

1. A new hybrid high-performance deep fake face detection method is used based on the analysis of the Fisher face algorithm (LBHH) with dimensional reduction in features of the face image.
2. To detect the fake and real image using deepfake detection classifier based on DBN with the RBM technique.

The paper has been organized as follows: section 2 describes about the review of literature, section 3 introduces deep fake detection using FF-LBPH-DBN, section 4 explains about the experimental results and section 5 concludes the paper with future directions.

## Related works

Deep fakes in the face manipulation are the frightening thing to distort the original facts in the digital images. In the advancement of technologies, deep fake detection of algorithms are necessary to be used these days in verifying the content of digital manipulation information. By using deep learning algorithms such as Generative Adversarial Neural Networks (GANs) are based on the concept of auto encoders and decoders for the implementation of detecting the fake images or videos (Yadav & Salmani, 2019). Deep fakes which include swapping of face images are carried out without the knowledge of the celebrities. It is also used to misrepresent the face images of the politicians. At first, swapping of face image was done in the photo of Abraham Lincoln (Badale et al., 2018). Yang, Li & Lyu (2019) have proposed a model to detect deep fake using head poses inconsistency. By using that model, the faces for various persons were created without modifying the original face expressions. Jagdale & Shah (2019) paper proposed an algorithm of NA-VSR for super resolution. The concept of the algorithm is that it reads the video and converts into frame by frame (Maheswaran et al., 2017). Then, the median filter is applied to remove the unnecessary noise in the video. By the use of bicubic interpolation technique, the density of the pixel in the image gets increased. Bicubic transformation is applied for the enhancement of the image. Yadav & Salmani (2019) have described the working principle of the deep fake techniques along with swapping of face images in a high precision value (Maheswaran et al., 2018). Generative Adversarial Neural Networks (GANs) contain two neural networks; one is generator and the other is discriminator. In the generator neural networks, the fake images are created from the given data set. At the same time, discriminator neural networks are used to evaluate the images which are synthesized by the generator and check its authenticity. The important problems of deep fake are so harmful due to defamation of individual character and assassination and spreading fake news in the society.

There are many such issues in the existing approaches in terms of inefficiency in detecting the deep fake images, high error rate, high consumption time also high and inaccuracy in accessing the data. This work FF-LBPH-DBN focuses mainly on the minimization of computation and the application for various metrological parameters in an efficient way. Table 1 shows the survey based on detection of the fake images (Vivek et al., 2018).

**Table 1:** Survey of deepfake algorithms.

Author name	Name of the method	Classifier	Data set
Guarnera, Giudice & Battiato (2020)	Pipeline Features uisng GAN	k-NN, SVM, LDA	Own (AttGAN, GDWCT, StarGAN, StyleGAN, StyleGAN2)
Neves et al. (2020)	Deep Learning	CNN	100K-Faces (StyleGAN)iFakeFaceDB
Dang et al. (2020)	Deep Learning	fusion of CNN with Attention Mechanism	DFFD (ProGAN, StyleGAN)
Hulzebosch, Ibrahimi & Worring (2020)	Deep Learning	CNN, AE	StarGAN, Glow, ProGAN, StyleGAN
Chen et al. (2020)	Deep Learning	CNN, LSTM	UADFV, Celeb-DF
Ranjan, Patil & Kazi (2020)	Deep Learning	CNN, LSTM	FaceForensics++, Celeb-DF, DeepFake Detection Challenge
Wang et al. (2019)	GAN-Pipeline	SVM	(InterFaceGAN, StyleGAN)
Nataraj et al. (2019)	Steganalysis	CNN	100K-Faces (StyleGAN)
Yu, Davis & Fritz (2018)	Deep Learning	CNN	(ProGAN, SNGAN, CramerGAN, MMDGAN)
Marra et al. (2019)	Deep Learning	CNN	(CycleGAN, ProGAN,Glow, StarGAN, StyleGAN)
Mahendhiran & Kannimuthu (2018)	Deep Learning	KNN, navie bayes, Neura network, random forest	Multimodal sentimental prediction
Arunkumar & Kannimuthu (2020)	Evolutionary model	Bird eye view methods	Data analytics

## Methodology

**Figure 1:**
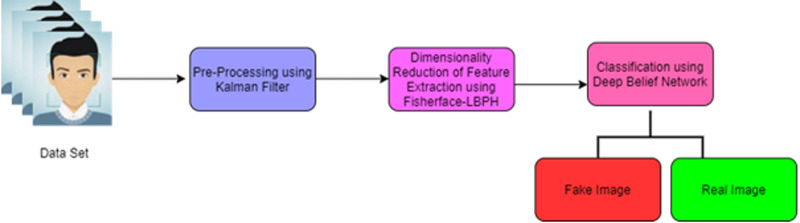
Architecture of proposed work.

The proposed face recognition and fake detection is based on the deep learning technique of the fisherface using the Local Binary Pattern Histogram (FF-LBPH). The accurate detection of deep fake image system consists of four phases such as (i) pre-processing, (ii) dimensionality reduction of image (iii) feature extraction and (iv) classification. This architecture of proposed work diagram is given in Fig. 1.

Figure 1 shows the pre-processing phase which includes resizing of images, removal of noise and normalization. For the improvement of feature extraction and classification process, the dimensionality reduction of face image is used by the fisherface algorithm (LBPH).

## Pre-Processing

**Figure 2:**
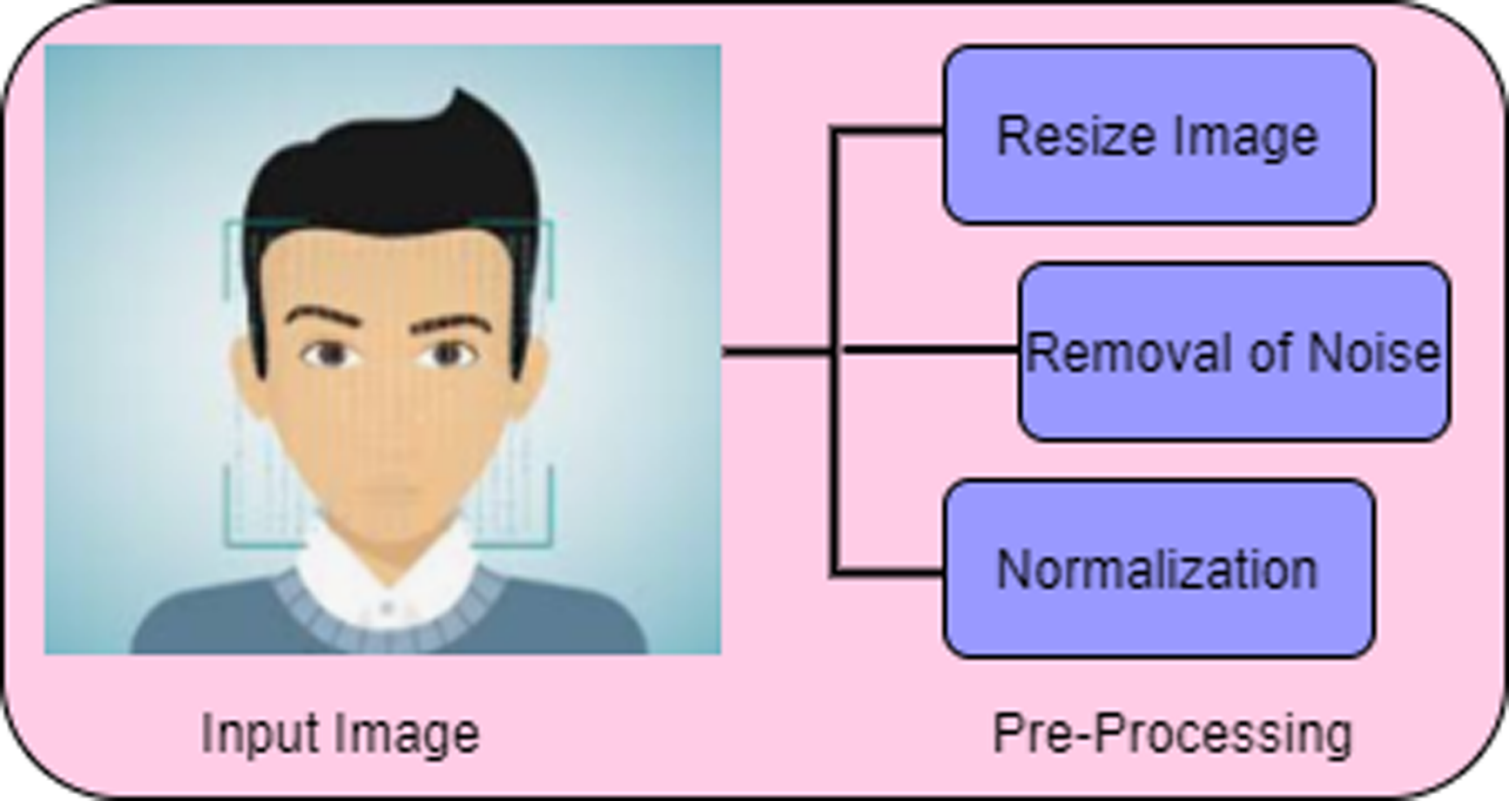
Pre-processing.

For classifying the deep fake image pre-processing is needed which enhancing the image for further processing. The steps involved in the phase of pre-processing are given in Fig. 2.

Figure 2 shows the three stages of pre-processing namely, resizing of image, removal of noise and normalization.

### Resize image

In the data set, all the images were in various sizes and the processing of various size data could not provide accurate result. All the images were resized as 256 × 256 and it was used for further processing. For resizing the input image downsampling and upsampling methods were employed.

### Removal of noise

In order to improve the efficiency in the classification of deep fake images, the noise was removed from raw input face image by using Kalman filter. Generally, it is a recursive mathematical model and it consists of two different processes; the prediction process and the update process. In the prediction process, priori system state is estimated from the previous state. And in the update process, the posteriority state is determined with the correction of priori state. The initial estimate state ${x}_{0}^{-}$ is repeated until the filtering process ends (Kalman, 1960; Arasaratnam, Haykin & Hurd, 2010). The Kalman filtering looping state is shown in Table 2.

**Table 2:** Filtering loops.

Prediction process	Update process
The priori estimate is calculated in the prediction process by using Eqn. ${\hat {X}}_{n}^{-}={T}_{n.}{\hat {X}}_{n-1}^{+}$	Kalman Gain matrix is represented using Eqn ${KG}_{n}={P}_{n}^{-}.{O}_{n}^{t}.({O}_{n}.{P}_{n}^{-}.{H}_{n}^{t}+{OUN}_{n})$
The covariance matrix is calculated using Eqn ${C}_{n}^{-}={T}_{n}.{P}_{n-1}^{+}.{T}_{n}^{t}+{PPN}_{n}$	The posteriori estimate is evaluated by using the Eqn ${Z}_{n}:{\hat {X}}_{n}^{+}={\hat {X}}_{n}^{-}+{KG}_{n}.({Z}_{n}-{O}_{n.}{\hat {X}}_{n}^{-})$
	Posteriori estimate covariance matrix is calculated as in Eqn ${P}_{n}^{+}= \left( I-{KG}_{n}.{O}_{n} \right) .{P}_{n}^{-}$

The parameters of Kalman filter are necessary to tune with covariance matrices of noises such as PPN, OUN and ${P}_{0}^{+}$. These covariance matrices are used to predict the weights. The noise of this filter has the zero multivariate Gaussian distribution of these covariance matrices. The covariance matrix of the sample vector *X* = [*X*_1,_*X*_2_, ..*X*_*n*_]^*T*^ is represented in Equation 1.

(1) \begin{eqnarray*}\sum = \left( \begin{array}{@{}ccc@{}} \displaystyle \sum _{1,1}&\displaystyle \cdots &\displaystyle \sum _{1,n}\\ \displaystyle \vdots &\displaystyle \ddots &\displaystyle \vdots \\ \displaystyle \sum _{n,1}&\displaystyle \cdots &\displaystyle \sum _{n,n} \end{array} \right) \end{eqnarray*}

where ${\sum }_{i,j}=cov \left( {X}_{i},{X}_{j} \right) =E \left( {X}_{i}-{\eta }_{i} \right) \left( {X}_{j}-{\eta }_{j} \right) ,~{\eta }_{i}=E[{X}_{i}]$, and where *E* is the expectation operator. The filter tuning is address with two approaches such as static and dynamic. The static tuning tunes the filter before the usage of it with the techniques such as autocovariance least squares (ALS). And the dynamic tuning tunes the filter while it is operating with self-tuning. Moreover, it uses the method called Artificial Neural Network. Once the data are pre-processed using the Kalman filter, the pre-processed data are then given as input to the feature selection phase in order to select the relevant features for classification.

### Normalization

For the enhancement, the contrast of image was used by using normalization. It was carried out based on pixel intensity value. The normalization process of this proposed work had used RGB pixel compensation method. It was based on the adaptive illumination of compensation dependent on the black pixel with histogram equalization.

## Dimensionality reduction using proposed fisherface-LBPH

Dimensionality reduction is an important step to reduce the dimension of the input image into low dimensional space. The proposed work was based on fusion of fisherface with Local Binary Pattern Histogram (FF-LBPH) which was utilized in the reduction of face space dimension. The fusion of fisherface with Linear Binary Pattern Histogram (LBPH) was implemented in the proposed study.

## Fisherface

The particular technique is based on Fisher’s Linear Discriminant Analysis (FLDA). The main advantage of the fisherface algorithm is faster in execution when compared to the eigenface technique. It is prominent for low error rates and also it works efficiently in various illuminations with different facial expressions. Steps involved in the fisherface algorithm are given below,

——————————————————————————————————

**Algorithm 1: Dimensionality reduction in feature extraction Fisher face (Proposed)**

——————————————————————————————————

**Input: Face Image from the data set**

**Output: Dimensionality reduction in feature extraction**

**Step 1**: Assume that size of the square face image with *height* = *width* = *N* and *img* is the number of images in the database.

**Step 2**: Select sample images form the database $ \left\{ \overrightarrow {a},\overrightarrow {b},~\ldots ,~\overrightarrow {e} \right\} $ and class scatter $c= \left\{ {x}_{1},{x}_{2},~\ldots ,~{x}_{n} \right\} $

face image 1 = $ \left\{ {\scriptsize \begin{array}{@{}c@{}} \displaystyle {\scriptsize \begin{array}{@{}c@{}} \displaystyle {a}_{1}\\ \displaystyle {a}_{2} \end{array}}\\ \displaystyle {a}_{3}\\ \displaystyle \vdots \\ \displaystyle {a}_{{N}^{2}} \end{array}} \right\} $; face image 2 = $ \left\{ {\scriptsize \begin{array}{@{}c@{}} \displaystyle {\scriptsize \begin{array}{@{}c@{}} \displaystyle {b}_{1}\\ \displaystyle {b}_{2} \end{array}}\\ \displaystyle {b}_{3}\\ \displaystyle \vdots \\ \displaystyle {b}_{{N}^{2}} \end{array}} \right\} ;$face image 3 = $ \left\{ {\scriptsize \begin{array}{@{}c@{}} \displaystyle {\scriptsize \begin{array}{@{}c@{}} \displaystyle {c}_{1}\\ \displaystyle {c}_{2} \end{array}}\\ \displaystyle {c}_{3}\\ \displaystyle \vdots \\ \displaystyle {c}_{{N}^{2}} \end{array}} \right\} $;

face image 4 = $ \left\{ {\scriptsize \begin{array}{@{}c@{}} \displaystyle {\scriptsize \begin{array}{@{}c@{}} \displaystyle {d}_{1}\\ \displaystyle {d}_{2} \end{array}}\\ \displaystyle {d}_{3}\\ \displaystyle \vdots \\ \displaystyle {d}_{{N}^{2}} \end{array}} \right\} $; face image 5 = $ \left\{ {\scriptsize \begin{array}{@{}c@{}} \displaystyle {\scriptsize \begin{array}{@{}c@{}} \displaystyle {e}_{1}\\ \displaystyle {e}_{2} \end{array}}\\ \displaystyle {e}_{3}\\ \displaystyle \vdots \\ \displaystyle {e}_{{N}^{2}} \end{array}} \right\} $; face image 6 = $ \left\{ {\scriptsize \begin{array}{@{}c@{}} \displaystyle {\scriptsize \begin{array}{@{}c@{}} \displaystyle {f}_{1}\\ \displaystyle {f}_{2} \end{array}}\\ \displaystyle {f}_{3}\\ \displaystyle \vdots \\ \displaystyle {f}_{{N}^{2}} \end{array}} \right\} $

**Step 3:** Calculate the average of all faces by using:

(2) \begin{eqnarray*}\overrightarrow {m}= \frac{1}{img} \left[ \begin{array}{@{}c@{}} \displaystyle {a}_{1}+{b}_{1}+\cdots +{f}_{1}\\ \displaystyle {a}_{2}+{b}_{2}+\cdots +{f}_{2}\\ \displaystyle \vdots ~~~~~~~\vdots ~~~~~~\vdots \\ \displaystyle {a}_{{N}^{2}}+{b}_{{N}^{2}}+\cdots +{f}_{{N}^{2}} \end{array} \right] \end{eqnarray*}

where *img* = 6

**Step 4:** Calculate the average face of each image in the data set

${\overrightarrow {img}}_{1}= \frac{1}{2} \left[ {\scriptsize \begin{array}{@{}c@{}} \displaystyle {a}_{1}+{b}_{1}\\ \displaystyle {a}_{2}+{b}_{2}\\ \displaystyle \vdots ~~~~~~\vdots \\ \displaystyle {a}_{{N}^{2}}+{b}_{{N}^{2}} \end{array}} \right] $; ${\overrightarrow {img}}_{2}= \frac{1}{2} \left[ {\scriptsize \begin{array}{@{}c@{}} \displaystyle {c}_{1}+{d}_{1}\\ \displaystyle {c}_{2}+{d}_{2}\\ \displaystyle \vdots ~~~~~~\vdots \\ \displaystyle {c}_{{N}^{2}}+{d}_{{N}^{2}} \end{array}} \right] $; ${\overrightarrow {img}}_{3}= \frac{1}{2} \left[ {\scriptsize \begin{array}{@{}c@{}} \displaystyle {e}_{1}+{f}_{1}\\ \displaystyle {e}_{2}+{f}_{2}\\ \displaystyle \vdots ~~~~~~\vdots \\ \displaystyle {e}_{{N}^{2}}+{f}_{{N}^{2}} \end{array}} \right] $**Step 5:** Subtract the average face of each image from the training images

(3) \begin{eqnarray*}{\overrightarrow {img}}_{1m}~= \left[ \begin{array}{@{}c@{}} \displaystyle {a}_{1}-{img}_{11}\\ \displaystyle {a}_{2}-{img}_{12}\\ \displaystyle \vdots ~~~~~~~~\vdots \\ \displaystyle {a}_{{N}^{2}}-{img}_{1{N}^{2}} \end{array} \right] ;{\overrightarrow {img}}_{2m}= \left[ \begin{array}{@{}c@{}} \displaystyle {b}_{1}-{img}_{11}\\ \displaystyle {b}_{2}-{img}_{12}\\ \displaystyle \vdots ~~~~~~~~\vdots \\ \displaystyle {b}_{{N}^{2}}-{img}_{1{N}^{2}} \end{array} \right] ;{\overrightarrow {img}}_{3m}= \left[ \begin{array}{@{}c@{}} \displaystyle {c}_{1}-{img}_{21}\\ \displaystyle {c}_{2}-{img}_{22}\\ \displaystyle \vdots ~~~~~~~~\vdots \\ \displaystyle {c}_{{N}^{2}}-{img}_{2{N}^{2}} \end{array} \right] \nonumber\\\displaystyle {\overrightarrow {img}}_{4m}= \left[ \begin{array}{@{}c@{}} \displaystyle {d}_{1}-{img}_{21}\\ \displaystyle {d}_{2}-{img}_{22}\\ \displaystyle \vdots ~~~~~~~~\vdots \\ \displaystyle {d}_{{N}^{2}}-{img}_{2{N}^{2}} \end{array} \right] ;{\overrightarrow {img}}_{5m}= \left[ \begin{array}{@{}c@{}} \displaystyle {e}_{1}-{img}_{31}\\ \displaystyle {e}_{2}-{img}_{32}\\ \displaystyle \vdots ~~~~~~~~\vdots \\ \displaystyle {e}_{{N}^{2}}-{img}_{3{N}^{2}} \end{array} \right] \nonumber\\\displaystyle ;{\overrightarrow {img}}_{6m}= \left[ \begin{array}{@{}c@{}} \displaystyle {f}_{1}-{img}_{41}\\ \displaystyle {f}_{2}-{img}_{42}\\ \displaystyle \vdots ~~~~~~~~\vdots \\ \displaystyle {f}_{{N}^{2}}-{img}_{4{N}^{2}} \end{array} \right] \end{eqnarray*}

**Step 6:** Create scatter matrix *sm*_1_,   *sm*_2_,  *sm*_3_, *sm*_4_

(4) \begin{eqnarray*}{sm}_{1}= \left( {\overrightarrow {img}}_{1m}{\overrightarrow {img}}_{1m}^{T}+{\overrightarrow {img}}_{2m}{\overrightarrow {img}}_{2m}^{T} \right) \end{eqnarray*}

(5) \begin{eqnarray*}{sm}_{2}= \left( {\overrightarrow {img}}_{3m}{\overrightarrow {img}}_{3m}^{T}+{\overrightarrow {img}}_{4m}{\overrightarrow {img}}_{4m}^{T} \right) \end{eqnarray*}

(6) \begin{eqnarray*}{sm}_{3}= \left( {\overrightarrow {img}}_{5m}{\overrightarrow {img}}_{5,m}^{T}+{\overrightarrow {img}}_{6m}{\overrightarrow {img}}_{6m}^{T} \right) \end{eqnarray*}

**Step 7:** Construct a scatter matrix within the class *s*_*mw*_ = *sm*_1_ + *sm*_2_ + *sm*_3_.

**Step 8:** Construct the scatter matrix between class

\begin{eqnarray*}{s}_{mb}=2 \left( {\overrightarrow {img}}_{1}-\overrightarrow {m} \right) { \left( {\overrightarrow {img}}_{1}-\overrightarrow {m} \right) }^{T}+2 \left( {\overrightarrow {img}}_{2}-\overrightarrow {m} \right) { \left( {\overrightarrow {img}}_{2}-\overrightarrow {m} \right) }^{T} \end{eqnarray*}

(7) \begin{eqnarray*}+2 \left( {\overrightarrow {img}}_{3}-\overrightarrow {m} \right) { \left( {\overrightarrow {img}}_{3}-\overrightarrow {m} \right) }^{T}\end{eqnarray*}

**Step 9:** Compute the vector *vec*_*img*_ and the columns of *vec*_*img*_ contain eigen vector values for ${s}_{mw}^{-1}{s}_{mb}$ . Here *s*_*mw*_ is minimized; *s*_*mb*_ is maximized by using:

(8) \begin{eqnarray*}{vec}_{img}= \left\vert \frac{{vec}_{img}^{T}{s}_{mb}}{{vec}_{img}^{T}{s}_{mw}} \right\vert \end{eqnarray*}

Generally, it can be defined by the decomposition of eigen value and it is represented as:

(9) \begin{eqnarray*}{s}_{mb}{vec}_{img}={s}_{mw}{vec}_{img}\mathrm{\Lambda }\end{eqnarray*}

Where, *vec*_*img*_ is eigen vector matrix and Λ are eigen values in the diagonal matrix. Eigen vectors *vec*_*img*_ are associated with eigen values of non-zero which are the fisherfaces.

**Step 10:** Normalization of the Equation

**Step 11:** Evaluate the weight for training image in the dataset in the normalized fisherface.

**Step 12:** Extracting features using dimensionality reduction of features so as to obtain face identification.

Algorithm 1 is an improvement version of eigen faces which include Principal Component Analysis (PCA) and Linear Discriminant Analysis (LDA). In order to get a sub-space and to maximize the variability within classes and between the classes of scatter matrix.

### Linear Binary Pattern Histogram (LBPH)

LBPH was used to recognize the facial images in the database. Extracting the features of face image and using binary operator, it recognized the image with less computational time complexity. Algorithm 2 describes LBPH.

—————————————————————————————————

**Algorithm 2: LBPH**

—————————————————————————————————

**Input: Input Face Image**

**Output: LBP pixel value**

**Step 1:** Split the face image into *n* × *n* (i.e) 8 × 8 which contains 64 parts or regions.

**Step 2:** Extraction of histogram values from each 64 sub-regions of face image using

(10) \begin{eqnarray*}{hist}_{i,j}=~\sum _{x,y}Img \left\{ {f}_{img} \left( x,y \right) =i \right\} ~Img \left\{ \left( x,y \right) \in {reg}_{j} \right\} \end{eqnarray*}

\begin{eqnarray*}where,~~i=0~to~n-1~\mathrm{{\XMLAMP}}~j=0~to~m-1 \end{eqnarray*}

m is the total number of sub-regions

n is the total number of class labels created by LBP operator.

**Step 3:** Apply Local binary operator in every sub-region and it is applied in 8 × 8 window size using

$LBP \left( i,j \right) ={\mathop{\sum }\nolimits }_{pix=0}^{pi-1}{2}^{pi}~su \left( {img}_{npix}-{img}_{c} \right) $ where, *i*, *j* is the centre pixel value of intensity *img*_*c*_, *img*_*npix*_ is the neighbour pixel value of intensity,

*su*  is the sub region of the image.

**Step 4:** Select pixel value of median as threshold value and compare it with neighbourhood pixel value of image 8 × 8 window size.

(11) \begin{eqnarray*}su \left( x \right) = \left\{ \begin{array}{@{}c@{}} \displaystyle 1,~x\geq 0\\ \displaystyle 0,~x\lt 0 \end{array} \right\} \end{eqnarray*}

If the neighbour pixel value is greater than or equal to the middle pixel value as 1, the value is set value as 0.

**Step 5:** Combine all the neighbour pixel values to form 8-bit binary number and convert it into decimal number and it is called as LBP pixel value. (range from 0-255).

In Algorithm 2, LBPH is the fusion of Local Binary Patterns (LBP) technique with Histograms of Oriented Gradients (HOG) descriptor. It is a simple and powerful method to extract the features and labelling the pixels in the face image.

### Dimensionality Reduction in feature extraction using proposed fisherface-LBPH

By reducing the dimension of face image and extracting the features using fusion of the fisherface algorithm with LBPH, the face image got recognized. The steps involved (FF-LBPH) are given below:

——————————————————————————————————

**Algorithm 3: Dimensionality reduction in feature extraction of fisherface-LBPH (Proposed)**

——————————————————————————————————

**Input: Face Image from the data set**

**Output: Recognizing the face image**

**Step 1:** Read face image of size *m* × *m* from the dataset and stored in form of column vector values.

**Step 2:** Normalize the input face training image and calculate the value of various matrix by subtracting the average value from the training image.

**Step 3:** Evaluate Algorithm 1 and extract the relevant features of face image.

**Step 4:** call algorithm 2

**Step 5:** Count the similar LBP pixel value in all sub region of face image.

**Step 6:** Combine all histogram value into single histogram value and it is stored as vector value for features of the face image.

**Step 7:** To recognize the similar images in the testing data set by performing the match process of testing image and calculating the minimum distance between original image and testing image using Euclidean distance:

(12) \begin{eqnarray*}dist \left( a,b \right) =\sum _{i=1}^{n} \left\vert \left\lfloor {histimg}_{1}-histimg2 \right\rfloor \right\vert \end{eqnarray*}

**Step 8:** Recognize the matching images.

In Algorithm 3, fusion of fisherface with LBPH is implemented. At first, it takes the image in same height and width. It extracts the features using the concept of principal components which differentiate one face image of individuality from the another. Therefore, each and every feature of the image cannot dominate the another. In order to obtain the characteristics of the face image features by reducing the face image space dimensions using fisher Linear discriminant (FDL) technique. After obtaining the features of face image,LBPH is applied in order to get a fine tune classification of deepfake face image. In method of LBPH, if the neighbourhood value is greater than the threshold (median value), it is taken as 1 else 0. Considering this value as a binary format, it is converted to a decimal format. Hence, this decimal format is called as LBP value. After generating the LBP value, the histogram of subregion is evaluated and the similar LBP values in the subregion of face image are counted. Then, all the histograms of subregion are merged to form a single histogram which is called as feature vector of the face image. By comparing the histogram of test face image with all images in the dataset, the closest histogram value of face image is recognized.

### Deepfake detection using classifier

#### Background of DeepFake

In the synthetic media, deepfakes are replacing the existing face image with image of someone else. It uses Generative Adversarial Networks (GANs) for manipulating the faces. The facial manipulation contains three phases namely face synthesis, face swap and facial attributes and expressions.

##### Face Synthesis.

Using GAN in this phase replaces the real face with the fake image. The best approach used in this phase is StyleGAN. In the StyleGAN unsupervised training process is implemented and it generates the images with variations such as hair, freckles, etc. It also enables the synthetic controls of the image.

##### Face swap.

In the face manipulation, face swap is one of the popular techniques. It is used to detect the image or video of a person fake or real after swapping its face in the image. The most popular database which contains real and fake videos are FaceForensics++. From the database, the fakevideos are used with the help of FaceSwap computer graphics concept and the other deep learning techniques such as DeepFakeFaceSwap.

##### Facial attributes and expressions.

Facial attribute modifications include color of skin, hair, age, gender. Similarly, change of face expressions include sad, happy, anger and so on. These are called as manipulation of facial attributes and its expressions. Moreover, the most popular mobile app is FaceApp. It uses StarGAN methos for performing the image-to image translation (Tolosana et al., 2020).

#### Deepfake detection using classifier of DBN-RBM

**Figure 3:**
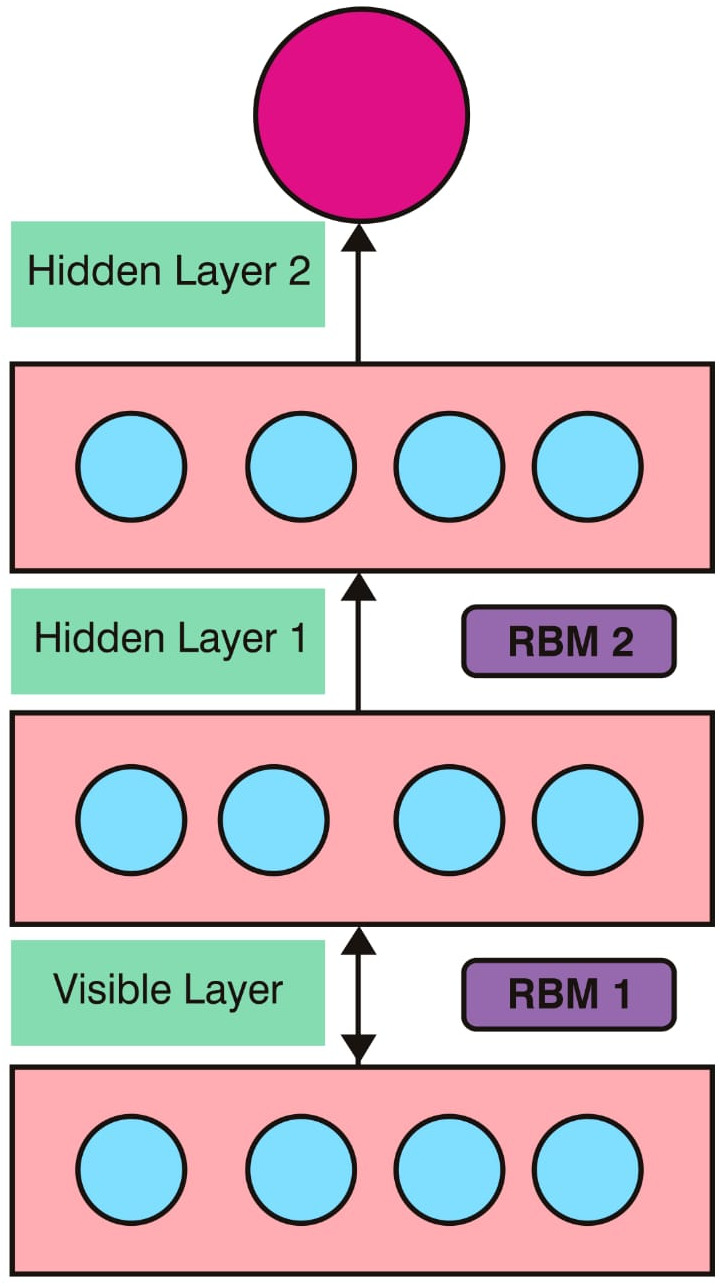
DBN with RBM.

Deepfake is a technique which uses the Generative Adversarial Networks (GANs) for generating fake images. Deepfake detection is based on the classifier algorithm Deep Belief Network (DBN) which is used to classify fake images from authentic image. DBN technique consists of three layers such as input, hidden and output layers. In addition to that, deep learning network contains stacked hidden layers and it is the extension of the neural network. DBN consists of one visible layer and multiple hidden layers. Transmission of input face image through visible layer to hidden layer is activated through sigmoid function based on the RBM learning rule (Hinton, Osindero & Teh, 2006). It is based on Restricted Boltzmann Machine (RBM) and DBN is acted with RBM which communicates with the previous layer and the subsequent layers in the DBN network. The architecture of DBN with RBM is shown in Fig. 3.

Figure 3 shows the architecture of DBN that consists of two stacked RBMs. RBM1 consists of visible layer and hidden layer 1, RBM2 consists of hidden layer 1 and hidden layer 2. In this architecture, the input face image is trained in the DBN based RBM classifier with the learning rule. The parameters which are used in the DBN architecture contain weight values between visible layer and hidden layer, value of bias and neuron states. Sigmoid function is applied for the transformation of neuron values from previous layer to the next layer using:

(13) \begin{eqnarray*}P \left( {sigmo}_{i}=1 \right) = \frac{1}{1+\mathrm{exp}\mathrm{}(-{b}_{i}-\sum _{j}{sigmo}_{j}{w}_{ij})} .\end{eqnarray*}

Bias and weight of all neurons are initialized in the RBM layer. In the training, the input face image consists of positive and negative phase. In the positive phase, input data is transformed from visible layer to hidden layer and negative phase transforms the input data from hidden layer to visible layer. In the positive and negative phases, individual activation function is calculated by using Eqn and that are defined as.

(14) \begin{eqnarray*}P({v}_{i}=1{|}h)=sigmo(-{b}_{i}-\sum _{j}{h}_{j}{w}_{ij})\end{eqnarray*}

(15) \begin{eqnarray*}P({h}_{i}=1{|}v)=sigmo(-{c}_{i}-\sum _{j}{h}_{j}{w}_{ij})\end{eqnarray*}

where, *v*_*i*_ isthe visible layer; *h*_*i*_ is the hidden layer and *w*_*ij*_ is the weight value.

This process is repeated and updated the weight value in the DBN architecture, until the maximum number of epochs is reached. The training process continued and the parametric values are optimized using:

(16) \begin{eqnarray*}update({w}_{ij}+ \frac{\eta }{2} \times \left( positive \left( {E}_{ij} \right) -negative \left( {E}_{ij} \right) \right) )\end{eqnarray*}

where,

$positive \left( {E}_{ij} \right) $-Positive statistics of edge *E*_*ij*_ = *p*(*h*_*j*_ = 1|*v*)

$negative \left( {E}_{ij} \right) $-Positive statistics of edge *E*_*ij*_ = *p*(*v*_*j*_ = 1|*h*)

*η*- learning rate

Using the above procedure, RBM is trained and the same process is repeated until all RBM get trained. By using the proposed work of dimensionality reduction in feature extraction, fisherface-LBPH was used for feature extraction with DBN-RBM classifier and it was used to recognize and differentiate the fake image and the real image.

## Result & Discussions

The proposed deep learning dimensionality reduction in feature extraction fisherface-LBPH evaluated the real and fake images in the public dataset. The public datasets used for deepfake detection were FFHQ, 100K-Faces, DFFD, CASIA-WebFace .

### Data set description

#### Flickr-Faces-HQ, FFHQ

Flickr-Faces-HQ, FFHQ dataset contains a group of 70,000 face images with a high-quality resolution generated by generative adversarial networks (GAN).

#### 100K-Faces

100K-Faces dataset contains 100,000 unique human face images generated using StyleGAN

#### Fake face dataset (DFFD)

DFFD dataset contains 100,000 and 200,000 fake images generated by ProGAN and StyleGAN. The dataset includes approximately 47.7 percent of male images, 52.3 percent of female images, and most of the sample images are in the range of age from 21 to 50 years old.

#### CASIA-WebFace

CASIA-WebFace database contains 10,000 subjects and 500,000 images. These images are crawled from IMDB website which has 10,575 of a well-known actors and actresses of IMDB.

### Performance metric measures

Performance metric measures such as accuracy and error detection rate are evaluated. To determine the performance of the proposed algorithm, it is compared with the existing approaches such as Supprot Vector Machine (SVM), LDA , KNN and Convolution Neural Network (CNN) . In order to evaluate the performance metric measures, accuracy, sensitivity, specificity, error rate of the Root Mean Square Error (RMSE), Signal-to-Noise-Ratio (SNR), Peak Signal-to-Noise-Ratio (PSNR), and Mean Absolute Error (MAE) are utilized.

### Accuracy

(17) \begin{eqnarray*}accuracy= \frac{TP+TN}{TP+TN+FP+FN} X100\end{eqnarray*}

### Sensitivity

(18) \begin{eqnarray*}Sensitivity= \frac{TP}{TP+FN} X100\end{eqnarray*}

### Specificity

It is used to evaluate the rate between True Negative (TN) and True Positive (TP)

(19) \begin{eqnarray*}Specificty= \frac{TN}{TN+FP} X100\end{eqnarray*}

The error rate is given below:

(20) \begin{eqnarray*}PSNR=20{\mathrm{log}}_{10} \left( \frac{{255}^{2}}{MAE} \right) ~\end{eqnarray*}

(21) \begin{eqnarray*}MAE= \frac{1}{MN} \sum _{i=1}^{M}\sum _{j=1}^{N} \left\vert X \left( i,j \right) -Y \left( i,j \right) \right\vert \end{eqnarray*}

(22) \begin{eqnarray*}RMSE=\sqrt{ \frac{1}{N} }\sum _{i=1}^{N}{ \left( {X}_{i}-\widehat{{X}_{i}} \right) }^{2}\end{eqnarray*}

(23) \begin{eqnarray*}SNR \left( db \right) =20log \left( \frac{{V}_{RMS(Signal)}}{{V}_{RMS(Noise)}} \right) \end{eqnarray*}

Table 2 shows that accuracy rate of proposed work that is compared with the different data set.

Table 3 shows the accuracy of various algorithm with the proposed work and it is implemented in various public available data set such as FFHQ, 100K-Faces, DFFD and CASIA-WebFace. The accuracy rate of proposed work FF-LBPH-DBN was high (98.82%) in the dataset of CASIA-WebFace image dataset. The next position in terms of accuracy rate is 97.82% for DFFD dataset. Table 4 shows error detection rate of proposed work in various data set.

**Table 3:** Performance comparison of proposed methods with different datasets in terms of accuracy.

Methods	Datasets			
	FFHQ	100K-Faces	DFFD	CASIA-WebFace
**SVM**	82.5	70.12	84.43	85.25
**LDA**	86.32	78.11	88.32	84.52
**KNN**	88.15	80.21	87.01	91.75
**CNN**	89.23	82.45	88.55	86.12
**Proposed FF-LBPH-DBN**	94.92	95.55	97.82	98.82

**Table 4:** Performance comparison of proposed methods with different datasets in terms of error detection rate.

Methods	Datasets			
	FFHQ	100K-Faces	DFFD	CASIA-WebFace
**SVM**	15.37	26.81	13.97	12.48
**LDA**	14.25	24.02	13.75	12.82
**KNN**	12.33	16.56	12.78	10.19
**CNN**	12.23	15.25	12.78	10.25
**Proposed FF-LBPH-DBN**	9.12	11.25	9.04	7.06

Table 4, shows the error rate of various algorithm in different data sets. The proposed work FF-LBPH-DBN got minimum error rate of 7.06 in the data set CASIA-WebFace data set. Table 5 shows the sensitivity, specificity performance comparison using various techniques namely SVM, LDA, KNN, CNN. The proposed work FF-LBPH-DBN with various datasets of FFHQ, 100K-Faces, DFFD, CASIA-WebFace were provided for better understanding.

**Table 5:** Sensitivity and specificity in different data sets.

Data Sets	Sensitivity %					Specificity %				
	SVM	LDA	KNN	CNN	FF-LBPH-DBN	SVM	LDA	KNN	CNN	FF-LBPH-DBN
**FFHQ**	85.3	796	83.9	82.4	89.67	82.75	85.7	81.45	86.78	91.22
**100K-Faces**	84.2	81	83.8	81.8	86.88	83.75	85.9	89.54	86.89	92.45
**DFFD**	82.9	86.3	82.7	80.9	88.9	86.75	86.9	88.45	84.56	93.76
**CASIA-WebFace**	89.2	81.2	89	87	91.35	85.78	88.4	85.12	87.91	94.35

Table 5 shows the sensitivity, specificity that provide best performance for FF-LBPH-DBN algorithm compared to the existing algorithms and various data sets of FFHQ, 100K-Faces, DFFD, CASIA-WebFace. Whereas, FF-LBPH-DBN of proposed work got sensitivity score as 89.67% in FFHQ data set, 86.88% in 100K-Faces data set, 88.9% in DFFD data set and 91.35% in CASIA-WebFace data set. Similarly for Specificity of proposed work FF-LBPH-DBN got 91.22% in FFHQ data set, 92.45% in 100K-Faces data set, 93.76% in DFFD data set and 94.35% in CASIA-WebFace data set. The deepfake detection of face image in the aspects of Equal Error Rate (EER) and AUC was done on the datasets of FFHQ, 100K-Faces, DFFD, CASIA-WebFace. These datasets were used in both training and testing process by deepfake detection classifier methods namely, deepfake, face swap, face synthesis with the proposed work of FF-LBPH-DBN model. Table 4 shows the performance of deepfake detection in the aspects of Equal Error Rate (EER) and AUC on various datasets.

**Figure 4:**
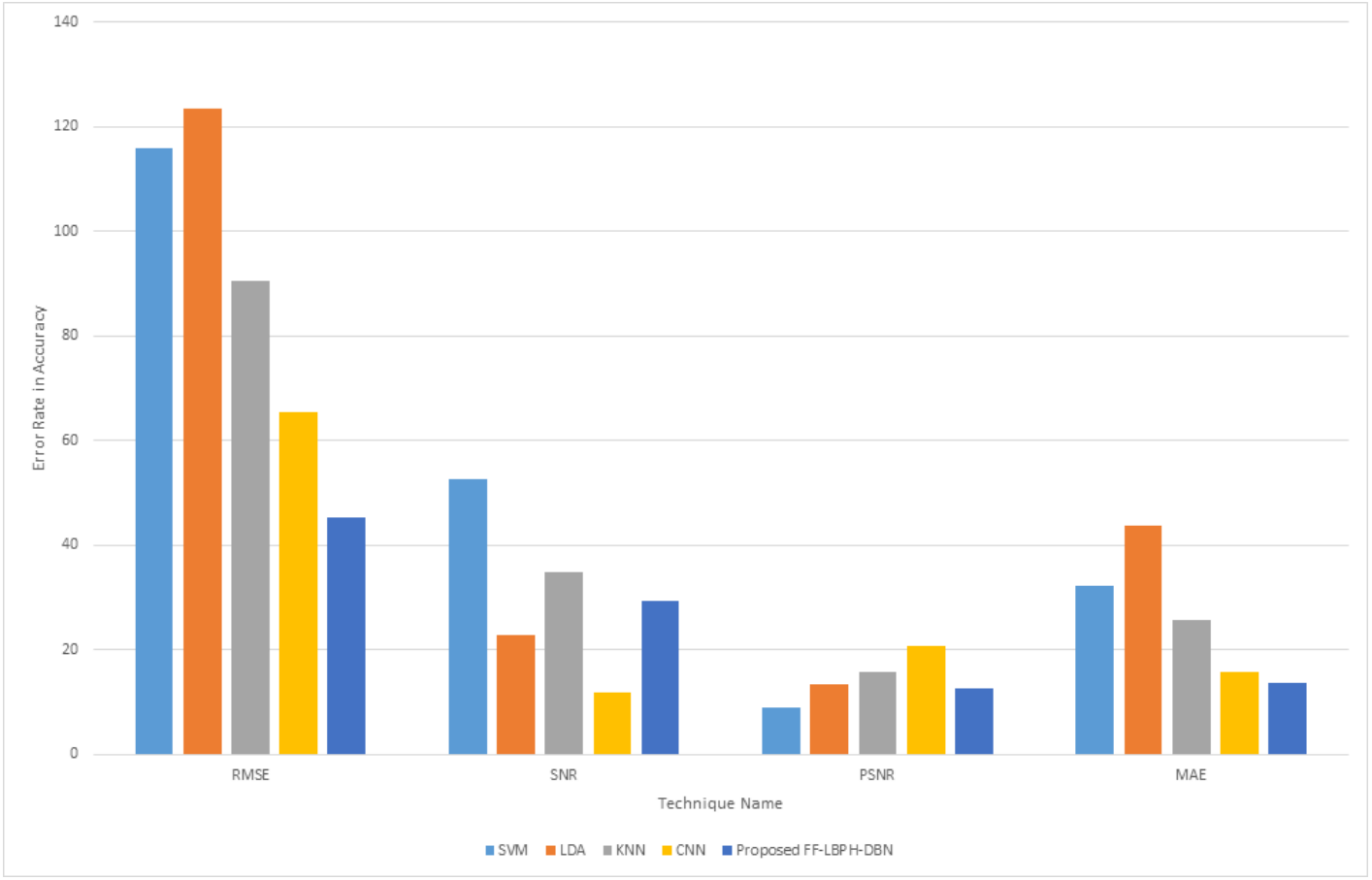
Error rate in accuracy.

Table 6 shows the exact recognition of real and fake images for deepfake, faceswap and face synthesis methods. In the proposed work, the dataset of CASIA-WebFace had attained better performance in the methods of deepfake with 0.978 in AUC and 7.21 in EER, faceswap with 0.986 in AUC and 9.56 in EER and facesynthesis with 0.788 in AUC and 12.32 in EER. Figure 4 shows the error rate value that is calculated based on its accuracy using the Eq22–Eq25.

**Table 6:** EER and AUC on deepfake detection methods.

Data Sets	Deepfake		Face swap		Face Synthesis	
	AUC	EER	AUC	EER	AUC	EER
**FFHQ**	0.948	13.33	0.918	13.65	0.726	16.49
**100K-Faces**	0.975	9.57	0.954	10.76	0.772	19.51
**DFFD**	0.969	12.45	0.944	12.78	0.714	15.67
**CASIA-WebFace**	0.978	7.21	0.986	9.56	0.788	12.32

**Figure 5:**
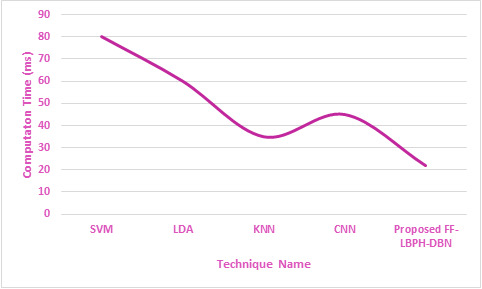
Computation time.

From Fig. 4, it is observed that PSNR value must be increased and MAE value must be decreased for the best detection of fake face image and real face image. The proposed approach provided better error rate value with the base of accuracy. In the proposed work the value of PSNR was increase and the value of MAE got decreased when compared to the other existing techniques. Figure 5 shows the computation time for various algorithms.

**Figure 6:**
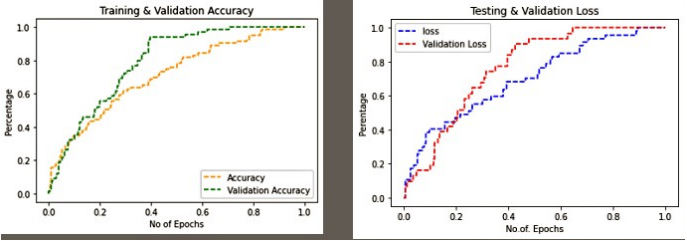
Training & validation accuracy and loss in proposed work.

In Fig. 5, it is revealed that the proposed algorithm of FF-LBPH-DBN needs less computation time when compared to the other existing algorithms. The analysis of training and testing of dataset in the face image had produced validation face input data in the terms of accuracy and loss metric information in the deepfake face image dataset. The proposed work of FF-LBPH-DBN model in epochs is shown in Fig. 6.

It is observed in Fig. 6 that the proposed methods FFHQ, 100K-Faces, DFFD, CASIA-WebFace of dataset provide less validation loss and good validation accuracy for FF-LBPH-DBN model.

## Conclusion

In this paper, the fisherface Linear binary pattern histogram using the DBN classifier (FF-LBPH DBN) technique was implemented as a detection technique for deepfake images. The proposed work was faster in execution and the detection of fake image and real image was very effective. Deepfake face image manipulations were analyzed using FF-LBPH DBN model and it also produced high level of accuracy. The pre-processing work had been done using Kalman filter for the detection of the fake images in a fine-tuned recognition. In order to get less execution of time, the dimensionality reduction of features were utilized using a fusion of the fisherface-LBPH algorithm. It helped in detecting the fake face image which in turn could prevent the individuals from being defamed unknowingly. From the results, it was concluded that the proposed work FF-LBPH had produced better detection and analysis of deepfake face image. The accuracy rate of proposed work FF-LBPH-DBN had attained a value of 98.82% in the CASIA-WebFace image dataset. The next position in terms of accuracy rate was 97.82% for the DFFD dataset. For future work, it may be extended up to various classifiers and use of different distance metric measures for detecting the deepfake face image.

## Supplemental Information

10.7717/peerj-cs.881/supp-1 Supplemental Information 1 Image dataset

10.7717/peerj-cs.881/supp-2 Supplemental Information 2 Code

## Additional Information and Declarations

### Data Availability

The following information was supplied regarding data availability: The data is available at Kaggle: https://www.kaggle.com/arnaud58/flickrfaceshq-dataset-ffhq.
