# Supplementary figures and images for: Deep learning model for deep fake face recognition and detection (part 2 of 2)
Source: PeerJ Comput Sci. 2022 Feb 22;8:e881. doi: 10.7717/peerj-cs.881 (PMC9044351; doi:10.7717/peerj-cs.881)

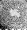

Supplement: Supplemental Information 1 [file peerj-cs-08-881-s001.zip › plant_1/plant_1_day_2_hour_15_depth.png]

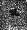

Supplement: Supplemental Information 1 [file peerj-cs-08-881-s001.zip › plant_1/plant_1_day_2_hour_15_depthSigma.png]

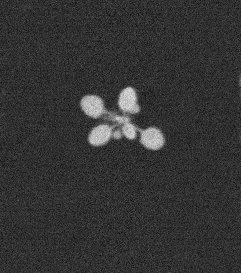

Supplement: Supplemental Information 1 [file peerj-cs-08-881-s001.zip › plant_1/plant_1_day_2_hour_15_fmp.png]

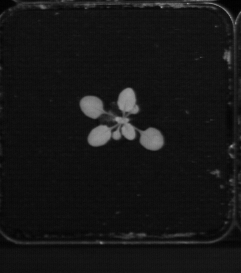

Supplement: Supplemental Information 1 [file peerj-cs-08-881-s001.zip › plant_1/plant_1_day_2_hour_15_ir.png]

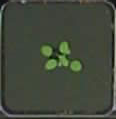

Supplement: Supplemental Information 1 [file peerj-cs-08-881-s001.zip › plant_1/plant_1_day_2_hour_15_rgb.png]

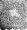

Supplement: Supplemental Information 1 [file peerj-cs-08-881-s001.zip › plant_1/plant_1_day_2_hour_16_depth.png]

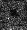

Supplement: Supplemental Information 1 [file peerj-cs-08-881-s001.zip › plant_1/plant_1_day_2_hour_16_depthSigma.png]

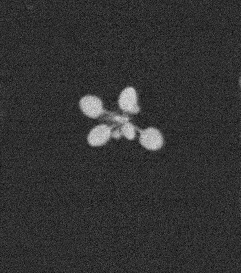

Supplement: Supplemental Information 1 [file peerj-cs-08-881-s001.zip › plant_1/plant_1_day_2_hour_16_fmp.png]

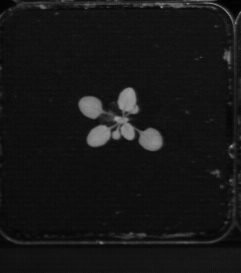

Supplement: Supplemental Information 1 [file peerj-cs-08-881-s001.zip › plant_1/plant_1_day_2_hour_16_ir.png]

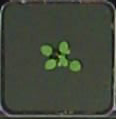

Supplement: Supplemental Information 1 [file peerj-cs-08-881-s001.zip › plant_1/plant_1_day_2_hour_16_rgb.png]

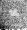

Supplement: Supplemental Information 1 [file peerj-cs-08-881-s001.zip › plant_1/plant_1_day_2_hour_17_depth.png]

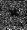

Supplement: Supplemental Information 1 [file peerj-cs-08-881-s001.zip › plant_1/plant_1_day_2_hour_17_depthSigma.png]

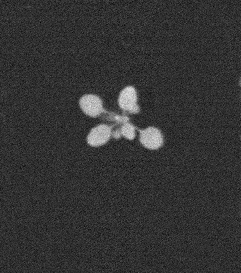

Supplement: Supplemental Information 1 [file peerj-cs-08-881-s001.zip › plant_1/plant_1_day_2_hour_17_fmp.png]

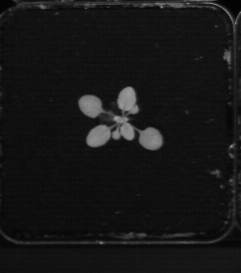

Supplement: Supplemental Information 1 [file peerj-cs-08-881-s001.zip › plant_1/plant_1_day_2_hour_17_ir.png]

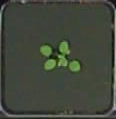

Supplement: Supplemental Information 1 [file peerj-cs-08-881-s001.zip › plant_1/plant_1_day_2_hour_17_rgb.png]

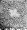

Supplement: Supplemental Information 1 [file peerj-cs-08-881-s001.zip › plant_1/plant_1_day_2_hour_18_depth.png]

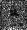

Supplement: Supplemental Information 1 [file peerj-cs-08-881-s001.zip › plant_1/plant_1_day_2_hour_18_depthSigma.png]

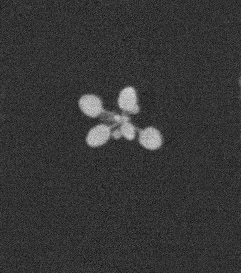

Supplement: Supplemental Information 1 [file peerj-cs-08-881-s001.zip › plant_1/plant_1_day_2_hour_18_fmp.png]

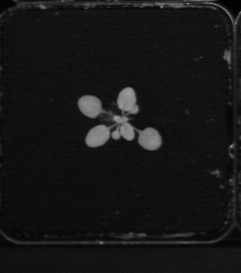

Supplement: Supplemental Information 1 [file peerj-cs-08-881-s001.zip › plant_1/plant_1_day_2_hour_18_ir.png]

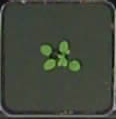

Supplement: Supplemental Information 1 [file peerj-cs-08-881-s001.zip › plant_1/plant_1_day_2_hour_18_rgb.png]

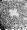

Supplement: Supplemental Information 1 [file peerj-cs-08-881-s001.zip › plant_1/plant_1_day_2_hour_19_depth.png]

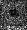

Supplement: Supplemental Information 1 [file peerj-cs-08-881-s001.zip › plant_1/plant_1_day_2_hour_19_depthSigma.png]

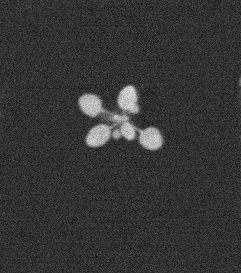

Supplement: Supplemental Information 1 [file peerj-cs-08-881-s001.zip › plant_1/plant_1_day_2_hour_19_fmp.png]

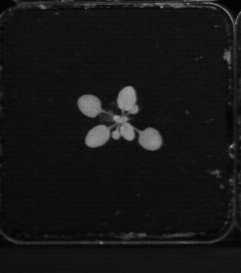

Supplement: Supplemental Information 1 [file peerj-cs-08-881-s001.zip › plant_1/plant_1_day_2_hour_19_ir.png]

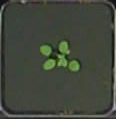

Supplement: Supplemental Information 1 [file peerj-cs-08-881-s001.zip › plant_1/plant_1_day_2_hour_19_rgb.png]

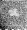

Supplement: Supplemental Information 1 [file peerj-cs-08-881-s001.zip › plant_1/plant_1_day_2_hour_20_depth.png]

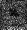

Supplement: Supplemental Information 1 [file peerj-cs-08-881-s001.zip › plant_1/plant_1_day_2_hour_20_depthSigma.png]

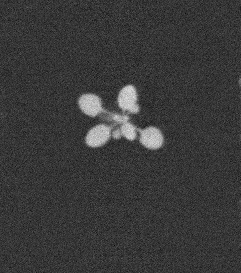

Supplement: Supplemental Information 1 [file peerj-cs-08-881-s001.zip › plant_1/plant_1_day_2_hour_20_fmp.png]

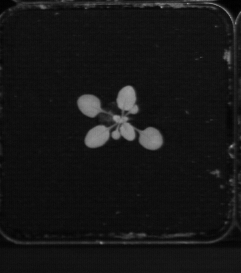

Supplement: Supplemental Information 1 [file peerj-cs-08-881-s001.zip › plant_1/plant_1_day_2_hour_20_ir.png]

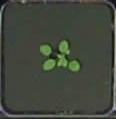

Supplement: Supplemental Information 1 [file peerj-cs-08-881-s001.zip › plant_1/plant_1_day_2_hour_20_rgb.png]

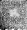

Supplement: Supplemental Information 1 [file peerj-cs-08-881-s001.zip › plant_1/plant_1_day_2_hour_21_depth.png]

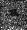

Supplement: Supplemental Information 1 [file peerj-cs-08-881-s001.zip › plant_1/plant_1_day_2_hour_21_depthSigma.png]

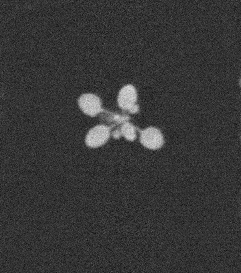

Supplement: Supplemental Information 1 [file peerj-cs-08-881-s001.zip › plant_1/plant_1_day_2_hour_21_fmp.png]

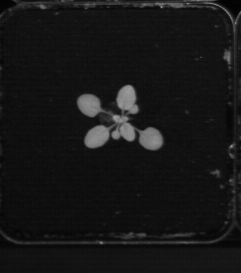

Supplement: Supplemental Information 1 [file peerj-cs-08-881-s001.zip › plant_1/plant_1_day_2_hour_21_ir.png]

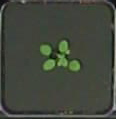

Supplement: Supplemental Information 1 [file peerj-cs-08-881-s001.zip › plant_1/plant_1_day_2_hour_21_rgb.png]

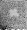

Supplement: Supplemental Information 1 [file peerj-cs-08-881-s001.zip › plant_1/plant_1_day_2_hour_22_depth.png]

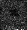

Supplement: Supplemental Information 1 [file peerj-cs-08-881-s001.zip › plant_1/plant_1_day_2_hour_22_depthSigma.png]

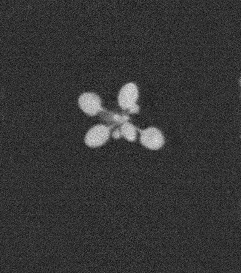

Supplement: Supplemental Information 1 [file peerj-cs-08-881-s001.zip › plant_1/plant_1_day_2_hour_22_fmp.png]

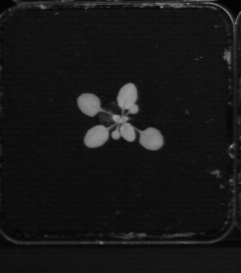

Supplement: Supplemental Information 1 [file peerj-cs-08-881-s001.zip › plant_1/plant_1_day_2_hour_22_ir.png]

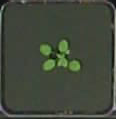

Supplement: Supplemental Information 1 [file peerj-cs-08-881-s001.zip › plant_1/plant_1_day_2_hour_22_rgb.png]

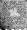

Supplement: Supplemental Information 1 [file peerj-cs-08-881-s001.zip › plant_1/plant_1_day_2_hour_23_depth.png]

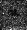

Supplement: Supplemental Information 1 [file peerj-cs-08-881-s001.zip › plant_1/plant_1_day_2_hour_23_depthSigma.png]

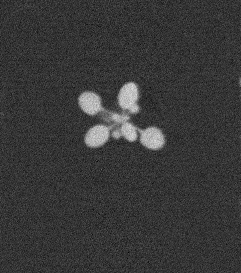

Supplement: Supplemental Information 1 [file peerj-cs-08-881-s001.zip › plant_1/plant_1_day_2_hour_23_fmp.png]

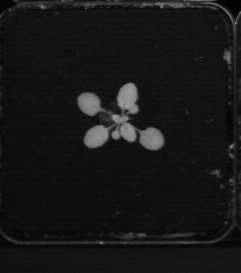

Supplement: Supplemental Information 1 [file peerj-cs-08-881-s001.zip › plant_1/plant_1_day_2_hour_23_ir.png]

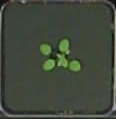

Supplement: Supplemental Information 1 [file peerj-cs-08-881-s001.zip › plant_1/plant_1_day_2_hour_23_rgb.png]

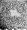

Supplement: Supplemental Information 1 [file peerj-cs-08-881-s001.zip › plant_1/plant_1_day_2_hour_9_depth.png]

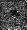

Supplement: Supplemental Information 1 [file peerj-cs-08-881-s001.zip › plant_1/plant_1_day_2_hour_9_depthSigma.png]

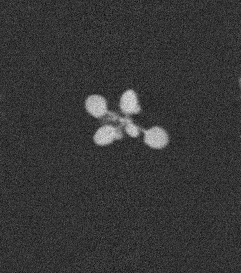

Supplement: Supplemental Information 1 [file peerj-cs-08-881-s001.zip › plant_1/plant_1_day_2_hour_9_fmp.png]

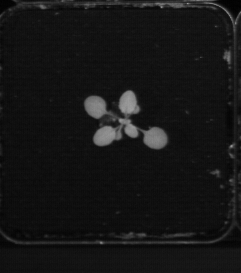

Supplement: Supplemental Information 1 [file peerj-cs-08-881-s001.zip › plant_1/plant_1_day_2_hour_9_ir.png]

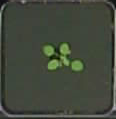

Supplement: Supplemental Information 1 [file peerj-cs-08-881-s001.zip › plant_1/plant_1_day_2_hour_9_rgb.png]

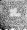

Supplement: Supplemental Information 1 [file peerj-cs-08-881-s001.zip › plant_1/plant_1_day_3_hour_10_depth.png]

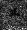

Supplement: Supplemental Information 1 [file peerj-cs-08-881-s001.zip › plant_1/plant_1_day_3_hour_10_depthSigma.png]

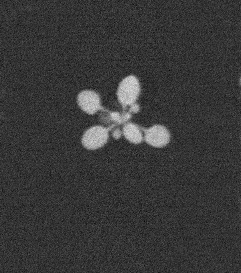

Supplement: Supplemental Information 1 [file peerj-cs-08-881-s001.zip › plant_1/plant_1_day_3_hour_10_fmp.png]

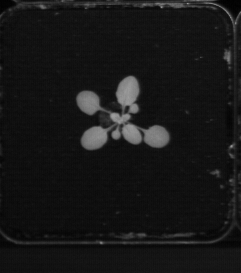

Supplement: Supplemental Information 1 [file peerj-cs-08-881-s001.zip › plant_1/plant_1_day_3_hour_10_ir.png]

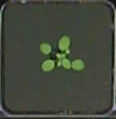

Supplement: Supplemental Information 1 [file peerj-cs-08-881-s001.zip › plant_1/plant_1_day_3_hour_10_rgb.png]

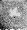

Supplement: Supplemental Information 1 [file peerj-cs-08-881-s001.zip › plant_1/plant_1_day_3_hour_11_depth.png]

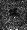

Supplement: Supplemental Information 1 [file peerj-cs-08-881-s001.zip › plant_1/plant_1_day_3_hour_11_depthSigma.png]

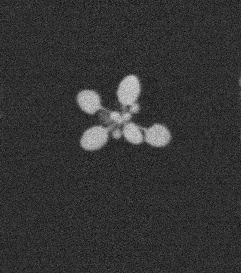

Supplement: Supplemental Information 1 [file peerj-cs-08-881-s001.zip › plant_1/plant_1_day_3_hour_11_fmp.png]

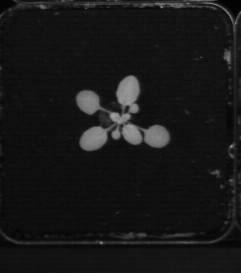

Supplement: Supplemental Information 1 [file peerj-cs-08-881-s001.zip › plant_1/plant_1_day_3_hour_11_ir.png]

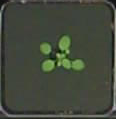

Supplement: Supplemental Information 1 [file peerj-cs-08-881-s001.zip › plant_1/plant_1_day_3_hour_11_rgb.png]

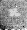

Supplement: Supplemental Information 1 [file peerj-cs-08-881-s001.zip › plant_1/plant_1_day_3_hour_12_depth.png]

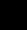

Supplement: Supplemental Information 1 [file peerj-cs-08-881-s001.zip › plant_1/plant_1_day_3_hour_12_depthSigma.png]

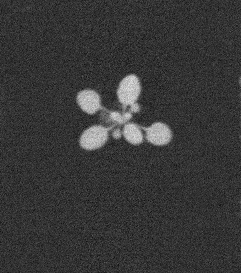

Supplement: Supplemental Information 1 [file peerj-cs-08-881-s001.zip › plant_1/plant_1_day_3_hour_12_fmp.png]

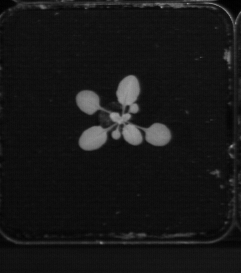

Supplement: Supplemental Information 1 [file peerj-cs-08-881-s001.zip › plant_1/plant_1_day_3_hour_12_ir.png]

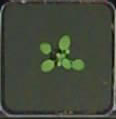

Supplement: Supplemental Information 1 [file peerj-cs-08-881-s001.zip › plant_1/plant_1_day_3_hour_12_rgb.png]

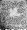

Supplement: Supplemental Information 1 [file peerj-cs-08-881-s001.zip › plant_1/plant_1_day_3_hour_13_depth.png]

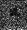

Supplement: Supplemental Information 1 [file peerj-cs-08-881-s001.zip › plant_1/plant_1_day_3_hour_13_depthSigma.png]

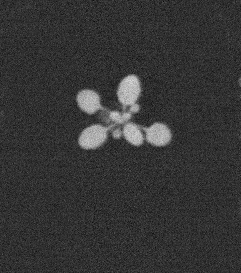

Supplement: Supplemental Information 1 [file peerj-cs-08-881-s001.zip › plant_1/plant_1_day_3_hour_13_fmp.png]

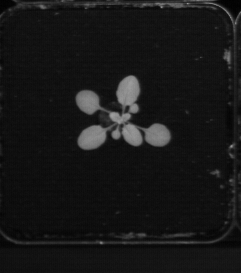

Supplement: Supplemental Information 1 [file peerj-cs-08-881-s001.zip › plant_1/plant_1_day_3_hour_13_ir.png]

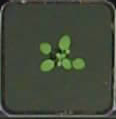

Supplement: Supplemental Information 1 [file peerj-cs-08-881-s001.zip › plant_1/plant_1_day_3_hour_13_rgb.png]

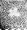

Supplement: Supplemental Information 1 [file peerj-cs-08-881-s001.zip › plant_1/plant_1_day_3_hour_14_depth.png]

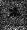

Supplement: Supplemental Information 1 [file peerj-cs-08-881-s001.zip › plant_1/plant_1_day_3_hour_14_depthSigma.png]

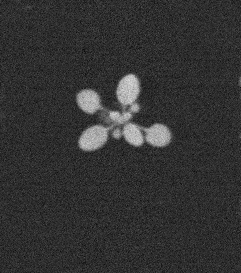

Supplement: Supplemental Information 1 [file peerj-cs-08-881-s001.zip › plant_1/plant_1_day_3_hour_14_fmp.png]

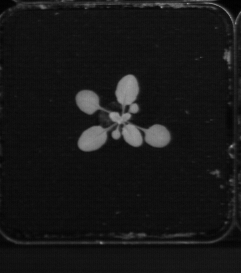

Supplement: Supplemental Information 1 [file peerj-cs-08-881-s001.zip › plant_1/plant_1_day_3_hour_14_ir.png]

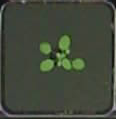

Supplement: Supplemental Information 1 [file peerj-cs-08-881-s001.zip › plant_1/plant_1_day_3_hour_14_rgb.png]

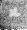

Supplement: Supplemental Information 1 [file peerj-cs-08-881-s001.zip › plant_1/plant_1_day_3_hour_15_depth.png]

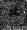

Supplement: Supplemental Information 1 [file peerj-cs-08-881-s001.zip › plant_1/plant_1_day_3_hour_15_depthSigma.png]

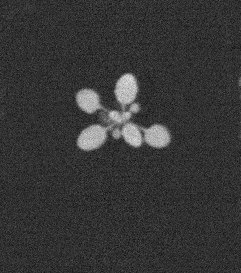

Supplement: Supplemental Information 1 [file peerj-cs-08-881-s001.zip › plant_1/plant_1_day_3_hour_15_fmp.png]

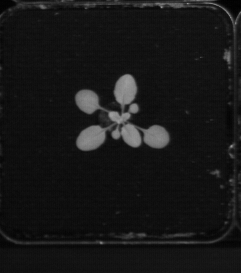

Supplement: Supplemental Information 1 [file peerj-cs-08-881-s001.zip › plant_1/plant_1_day_3_hour_15_ir.png]

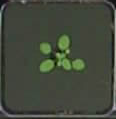

Supplement: Supplemental Information 1 [file peerj-cs-08-881-s001.zip › plant_1/plant_1_day_3_hour_15_rgb.png]

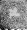

Supplement: Supplemental Information 1 [file peerj-cs-08-881-s001.zip › plant_1/plant_1_day_3_hour_16_depth.png]

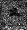

Supplement: Supplemental Information 1 [file peerj-cs-08-881-s001.zip › plant_1/plant_1_day_3_hour_16_depthSigma.png]

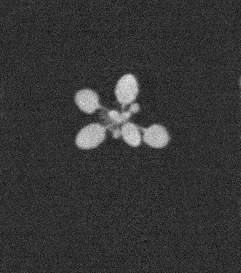

Supplement: Supplemental Information 1 [file peerj-cs-08-881-s001.zip › plant_1/plant_1_day_3_hour_16_fmp.png]

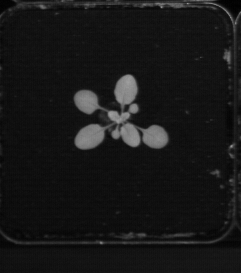

Supplement: Supplemental Information 1 [file peerj-cs-08-881-s001.zip › plant_1/plant_1_day_3_hour_16_ir.png]

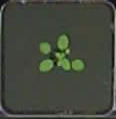

Supplement: Supplemental Information 1 [file peerj-cs-08-881-s001.zip › plant_1/plant_1_day_3_hour_16_rgb.png]

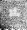

Supplement: Supplemental Information 1 [file peerj-cs-08-881-s001.zip › plant_1/plant_1_day_3_hour_17_depth.png]

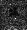

Supplement: Supplemental Information 1 [file peerj-cs-08-881-s001.zip › plant_1/plant_1_day_3_hour_17_depthSigma.png]

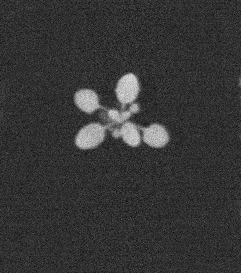

Supplement: Supplemental Information 1 [file peerj-cs-08-881-s001.zip › plant_1/plant_1_day_3_hour_17_fmp.png]

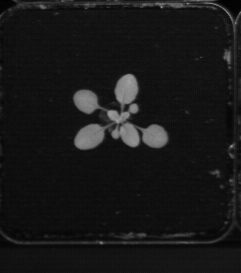

Supplement: Supplemental Information 1 [file peerj-cs-08-881-s001.zip › plant_1/plant_1_day_3_hour_17_ir.png]

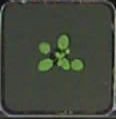

Supplement: Supplemental Information 1 [file peerj-cs-08-881-s001.zip › plant_1/plant_1_day_3_hour_17_rgb.png]

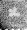

Supplement: Supplemental Information 1 [file peerj-cs-08-881-s001.zip › plant_1/plant_1_day_3_hour_18_depth.png]

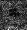

Supplement: Supplemental Information 1 [file peerj-cs-08-881-s001.zip › plant_1/plant_1_day_3_hour_18_depthSigma.png]

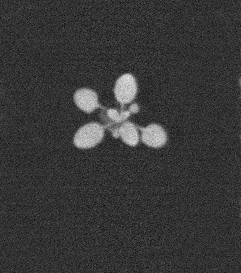

Supplement: Supplemental Information 1 [file peerj-cs-08-881-s001.zip › plant_1/plant_1_day_3_hour_18_fmp.png]

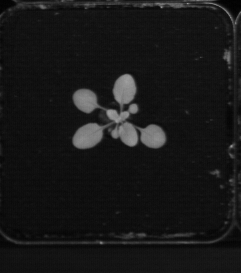

Supplement: Supplemental Information 1 [file peerj-cs-08-881-s001.zip › plant_1/plant_1_day_3_hour_18_ir.png]

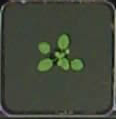

Supplement: Supplemental Information 1 [file peerj-cs-08-881-s001.zip › plant_1/plant_1_day_3_hour_18_rgb.png]

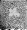

Supplement: Supplemental Information 1 [file peerj-cs-08-881-s001.zip › plant_1/plant_1_day_3_hour_19_depth.png]

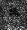

Supplement: Supplemental Information 1 [file peerj-cs-08-881-s001.zip › plant_1/plant_1_day_3_hour_19_depthSigma.png]

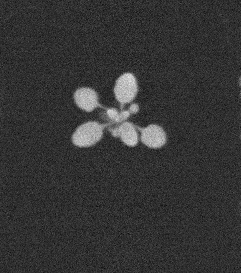

Supplement: Supplemental Information 1 [file peerj-cs-08-881-s001.zip › plant_1/plant_1_day_3_hour_19_fmp.png]

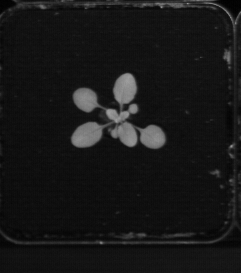

Supplement: Supplemental Information 1 [file peerj-cs-08-881-s001.zip › plant_1/plant_1_day_3_hour_19_ir.png]

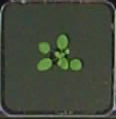

Supplement: Supplemental Information 1 [file peerj-cs-08-881-s001.zip › plant_1/plant_1_day_3_hour_19_rgb.png]
